# ENHANCE-D: protocol for a pragmatic, 3-arm, randomised controlled trial comparing the impact of enhanced smoking cessation interventions to very brief advice for adult smokers in dental care settings

**DOI:** 10.1186/s13063-025-08954-z

**Published:** 2025-07-28

**Authors:** Richard Holliday, Nina Wilson, Vicky Ryan, Chrissie Butcher, Tara Homer, Philippa Watts, Dorcas Kareithi, Paul Blaylock, Laura Ternent, Roland Finch, Susan M. Bissett, Adam Todd, Helen Hancock, Fiona Ellwood, David I. Conway, Nicholas S. Jakubovics, Ralf Kist, Richard D. Holmes, Linda Bauld, Philip M. Preshaw, Elaine McColl

**Affiliations:** 1 School of Dental Sciences, Faculty of Medical Sciences, Newcastle University, Newcastle Upon Tyne, UK; 2 Newcastle Upon Tyne Hospitals NHS Foundation Trust, Newcastle Upon Tyne, UK; 3 Population Health Sciences Institute, Faculty of Medical Sciences, Newcastle University, Newcastle Upon Tyne, UK; 4 Newcastle Clinical Trials Unit, Newcastle University, Newcastle Upon Tyne, UK; 5 ENHANCE-D Trial Patient Participant Group, Newcastle Upon Tyne, UK; 6 School of Pharmacy, Faculty of Medical Sciences, Newcastle University, Newcastle Upon Tyne, UK; 7 Society of British Dental Nurses/National Oral Health Promotion Group, Hitchin, UK; 8 School of Medicine, Dentistry, and Nursing, University of Glasgow, Glasgow, UK; 9 Usher Institute and Behavioural Research UK, University of Edinburgh, Edinburgh, UK; 10 School of Dentistry, University of Dundee, Dundee, UK

**Keywords:** Electronic nicotine delivery system (MESH), Smoking (MESH), Smoking cessation (MESH), Tobacco use cessation (MESH), Nicotine Replacement Therapy (MESH), Dental Health Services (MESH), Periodontal diseases (MESH), Periodontitis (MESH), Public Health Dentistry (MESH), Addictive behaviours (MESH)

## Abstract

**Background:**

Smoking is a key contributor to health inequalities, particularly impacting oral health. Periodontal (gum) health is significantly affected by smoking. With their extensive reach, regular and frequent patient contact, and potential teachable moments, dental teams are well placed to support patients to stop smoking. This is currently mainly provided through Very Brief Advice (VBA) interventions and so there is scope to enhance the cessation support offered. A large definitive trial is needed to confirm whether this would lead to improved smoking quit rates, improved oral health, and be good value for money.

**Methods:**

The ENHANCE-D trial is a multi-centre, pragmatic, definitive, 3-arm, parallel group, individually randomised controlled superiority trial, including an internal pilot, conducted in NHS dental settings. In total, 1215 patients who are current regular smokers will be randomly allocated using a 1:2:2 ratio to the following: (i) VBA; (ii) the offer of a standard course of Nicotine Replacement Therapy (NRT); (iii) or the offer of an e-cigarette starter kit. A subgroup of patients with periodontitis (gum disease) will have additional oral examinations and samples collected. The primary outcome is biochemically verified smoking abstinence at 6 months. A key secondary outcome is the percentage of periodontal sites with probing pocket depths (PPDs) ≥ 5 mm at 6 months in the periodontitis subgroup. Participants will be blinded to the purpose of the trial and not be aware of the treatment packages. Periodontal health outcomes will be recorded by trained and aligned assessors, blinded to intervention arm. Participant recruitment started in July 2022 with an internal pilot progressing to the main trial in May 2023. A qualitative process evaluation will explore participants’ experiences of receiving the study interventions, alongside the views of dental teams, NHS commissioners, service managers, and policymakers. An economic evaluation will include a cost-effectiveness analysis and a cost–benefit analysis.

**Discussion:**

This will be the largest contemporary randomised trial of smoking cessation interventions in dental settings and one of the first to evaluate enhanced interventions. E-cigarettes were included as an intervention due to growing evidence of their effectiveness. They will be compared to VBA and NRT, and oral health outcomes assessed. The multi-centre, pragmatic design of this trial supports the external validity and potential for impacting clinical practice.

**Trial registration:**

ISRCTN 13158982. Registered on 10 May 2022. https://www.isrctn.com/ISRCTNISRCTN13158982

**Supplementary Information:**

The online version contains supplementary material available at 10.1186/s13063-025-08954-z.

## Introduction

### Background and rationale

Smoking is a significant contributor to health inequalities, particularly affecting oral health and a major contributor to inequalities in the burden of oral diseases in the UK and globally [1, 2]. Dental settings, with their extensive reach and frequent patient contact, present a key opportunity to address these inequalities more effectively. Periodontitis is ranked as the sixth most common global health condition, affecting roughly 10% of UK adults and being a major cause of tooth loss [3]. The relationship between smoking and periodontitis is well-documented, with smoking not only increasing the risk but also impairing the effectiveness of treatments [4]. Smoking cessation support should be a core component of periodontal therapy. Similarly, smoking is a major risk factor for oral cancers with smoking cessation demonstrating a substantial risk reduction [5].

Research supports the benefits of quitting smoking for both oral health and treatment success [4, 6]; however, the optimal approach for supporting cessation in dental settings remains unclear [7]. Dental professionals in the UK are encouraged to offer Very Brief Advice (VBA) on smoking cessation [8]. In medical settings, VBA interventions typically increase the quit rate at 6 months from 3 to 5% (RR 1.66) [9]. Greater improvement in quit rates is seen with more intensive behavioural support and the addition of pharmacotherapy [10, 11]. Hence, the current approach of providing VBA alone in the dental setting is potentially a missed opportunity and there is considerable scope for enhancing the smoking cessation support offered.

There is high-quality evidence that Nicotine Replacement Therapy (NRT), particularly combination NRT, is effective in general populations motivated to quit [11, 12]. However, there is less evidence to support the use of NRT in dental settings and no studies conducted in UK primary dental care [7]. E-cigarettes (ECs) are a popular quit aid with growing evidence of effectiveness for smoking cessation [13, 14] although there is currently an evidence gap in non-specialist real-world settings such as dental settings. Evidence is also needed on any effects on oral health and treatment outcomes.

The ENHANCE-D trial is designed to build on current evidence and an external feasibility study with pilot RCT [15], by assessing the clinical and cost-effectiveness, as well as the safety, of introducing enhanced smoking cessation interventions (such as NRT and EC) in primary dental care settings. In addition, this trial aims to fill the gap in evidence concerning the effectiveness of ECs in non-specialist settings and also their potential impacts on oral health and treatment outcomes.

### Objectives

The primary objective is to compare biochemically verified smoking abstinence at 6 months of NRT and EC interventions to usual care (VBA) and to each other in adult regular tobacco smokers attending an NHS dental setting.

The key secondary objective is to compare the periodontal health at 6 months of NRT and EC interventions to usual care and to each other, for those with periodontitis at baseline. Other secondary objectives include assessing: smoking abstinence at 12 months, oral health, cost-effectiveness, patient quality of life, and intervention acceptability and associated experiences of the patients and dental professionals. We will also compare the occurrence of pre-specified AEs.

An 8-month internal pilot tested the feasibility of site recruitment and set-up, participant recruitment, and monitored the proportion of participants recruited to the periodontitis sub-study.

## Methods

In writing this paper, the SPIRIT reporting guidelines were followed (see Additional file 2: SPIRIT checklist) [16].

### Trial design

This trial is a pragmatic, definitive, 3-arm, parallel group, individually randomised controlled, superiority trial, including an internal pilot. Participants will be randomised in a 1:2:2 ratio between usual care (VBA) and the two interventions (offer of either standard NRT or EC starter kit). The main trial will include a sub-study in a subset of the participants with periodontitis at baseline.

### Internal pilot

At the end of the 8-month internal pilot (July 2022 to February 2023), we projected recruitment to be 365 participants and 56 sites. The internal pilot had a traffic light system (see Additional file 1). For amber, we needed to reach at least 50% of projected recruitment and open at least 50% of sites. We had recruited 167/365 (46%) participants and opened 30/56 (54%) sites by this point. After discussion with the funder, it was decided the trial would continue with stop/go criteria based on recruiting 545 participants and opening at least 15 more sites by the end of November 2023. By this date, we had recruited 699 participants and opened 42 sites (with several others in set up stages) and the funder approved continuation of the trial.

### Trial setting

The main trial will be completed in up to 56 NHS dental settings (primary care) across the UK. The dental settings were originally located within seven research regions around research hubs (the dental schools/institutes in Dundee, Edinburgh, Glasgow, Newcastle, Sheffield, Birmingham, and Plymouth), representing a diverse population with different NHS payment systems. Following the internal pilot, a further two research regions (South/South East, and East) were identified to extend coverage across a wider geographical area beyond the original hubs. The periodontitis sub-study will be conducted in primary care dental settings within reach of the original seven research hubs. Sites will be informed if they are able to take part in the periodontitis sub-study based on location and hub team availability.

### Participants

#### Inclusion criteria

The study included adults aged at least 18 years who are current regular smokers (self-identified, no criteria) with a Basic Periodontal Examination (BPE) completed within the last 3 months and willing to provide informed consent. Those in the periodontitis subgroup additionally require a minimum of 16 natural teeth (excluding the third molars) and a diagnosis of periodontitis stage II (or greater), grade A/B/C and currently unstable [17] (as diagnosed by the primary care dentist/dental hygienist/dental therapist).

#### Exclusion criteria

The following are excluded: known to be pregnant or currently breastfeeding, enrolled in another interventional research trial that could affect the outcome of this trial, currently enrolled on a formal programme to reduce/stop alcohol intake or stop smoking, phaeochromocytoma, uncontrolled hyperthyroidism, extensive dermatitis/skin disorder, known hypersensitivity to nicotine or any component of the study products and taking one or more of Clozapine, Olanzapine, Theophylline, Aminophylline.

### Recruitment

#### Participant Identification for the main study

ENHANCE-D will be conducted in any NHS primary care dental setting including general dental practices, community services, and undergraduate clinics in dental hospitals. Each setting is variable in its organisation and hence the recruitment strategy is flexible in line with the pragmatic design of this trial.

Participants will be identified by either examining dental records prior to the patient’s appointment and providing the information ahead of time, or opportunistically during their attendance at their dental visits. In most cases, it is anticipated that a two-visit approach will be taken where the dental professional will introduce the trial and provide the trial information at their routine visit. A second visit will be arranged to obtain informed consent, confirm eligibility, perform randomisation, and deliver the interventions. In some cases, it may be preferable to complete all the processes in one session.

A screening log will be kept which will include details of all patients who were provided with a participant information sheet and considered for trial participation. This log will include the reason that any patient did not take part, if applicable. Data (year of birth, sex, and postcode) from the screening log will be entered into the trial database.

A summary participant journey diagram is available in Additional File 1.

#### Patient identification for the periodontitis sub-study

For sites open to recruitment into the periodontitis sub-study, the primary care dental professionals will identify patients who are eligible for the sub-study while establishing eligibility for the main trial. When a potentially eligible patient is identified, contact will be made with the local hub team to arrange for baseline oral health data and samples to be collected. These will be collected before (or on the same day) as the interventions are delivered.

#### Who will take informed consent?

Participants will be allowed time to consider the patient information sheet (PIS), to decide whether they will participate in the trial, and will have the opportunity to ask any questions. Written informed consent will be received by delegated dental professionals who are General Dental Council registered, trained, and have been authorised to do so by the Principal Investigator. Participants will also have the option to consent to be contacted to take part in the qualitative interviews.

#### Additional consent provisions for collection and use of participant data and biological specimens

Participants in the periodontitis sub-study will receive a different PIS and informed consent form detailing the additional assessments involved. Periodontitis participants will provide written consent to the collection of samples (as detailed in the schedule of events) and can provide optional consent for these samples to be stored and used in future health-related research, including by commercial companies.

#### Randomisation

Following completion of informed consent and confirmation of eligibility, participants will be randomised to receive VBA, NRT, or EC starter kit, in a 1:2:2 ratio using random permuted blocks within strata. Randomisation will be stratified by research hub/region (up to 12 levels) and by baseline periodontal status (3 levels: (i) all sextants BPE code ≤ 2, (ii) 1–3 sextants with BPE code 3 or 4, (iii) ≥ 4 sextants with BPE code 3 or 4).

Randomisation will be performed by a member of trained site staff using the Sealed Envelope™ system (a secure, central, 24h web-based randomisation system which generates the allocation sequence with concealed allocation) [18].

### Trial Interventions

#### Oral health advice

All participants will be provided with a package of oral health advice, which is usual care in general dental practice. Dental professionals delivering the intervention in ENHANCE-D will utilise existing resources [19] to guide discussion through the topics of oral hygiene, fluoride, diet, alcohol, and smoking. The smoking aspect of this package of advice will vary depending on the randomisation group (VBA, NRT or EC).

#### Control (Very Brief Advice, VBA)

VBA is usual care for smokers in dental settings. A range of guidance documents recommend VBA, usually following the 3As (Ask, Advise, Act) technique. A recent survey reported 81% of dentists and 100% of dental hygienists and therapists provided this advice to smokers [20]. This intervention will signpost participants to a GP, pharmacy, or Stop Smoking Service (SSS). Formal referral to SSS can also be made where available. As this is a pragmatic trial, dental professionals will be asked to follow their usual practice in this regard.

Participants in the control group will be free to use NRT or ECs as they wish, but these will not be provided by the dental professional providing the advice, nor specifically recommended. It would be impractical and unethical to prohibit NRT or EC use given their widespread availability and because the GP or SSS might recommend or provide these as part of usual care. NRT and EC use will be recorded at follow-up for all participants. Likewise, use of any other smoking cessation medication/device was not prohibited in any of the groups.

#### Nicotine Replacement Therapy (NRT)

Participants randomised to the NRT group will be offered standard NRT. A trained dental professional will provide a single-visit behavioural support intervention, including the offer of NRT. Arrangements will be made for the supply of a 12-week course of combination NRT (nicotine patch plus faster-acting form such as chewing gum or lozenge), in line with current recommendations [21]. Stocks of NRT will be kept centrally at Newcastle Specials Pharmacy Production Unit (PPU) and not at each site. Each site is provided with demonstration versions of each product, and the dental professional will select the most appropriate products and strengths in consultation with the participant. These will be ordered by the site, prepared by the Newcastle Specials PPU and dispatched to each participant’s home via secure next day delivery or to the local dental practice for collection. Initially, a 4-week supply will be provided. During the third week, the dental professional will contact the participant via telephone or SMS text message to check on their status, and if required (e.g. they have quit or are still attempting to quit and need more supplies), they will order the further eight-week supply of NRT to be delivered to the patient’s home or dental practice. Participants are also provided with an NRT-specific PIS.

#### E-cigarette (EC) starter kit

Participants randomised to this group will be offered an EC starter kit. They will receive the same behavioural intervention as the NRT group. As with the NRT group, the EC starter kits will be stored centrally and shipped to the patient’s home address or local dental practice for collection. The starter kit will include ten x 10 ml bottles of e-liquid with a choice of one of four packages of flavour and nicotine concentrations. Further details of the EC starter kit are included in Additional file 1. The e-cigarette and liquids selected are MHRA-registered products. Each site will have demonstration models for training purposes. Participants will be expected to source their own supply of e-liquid after the initial supply; advice will be given as to where to source suitable MHRA-registered products. Participants will also be provided with an EC-specific PIS.

#### Single-visit smoking cessation behavioural intervention

Participants in the NRT and EC groups will be provided with a single-visit behavioural support intervention. Existing evidence suggests that pharmacological interventions (such as the NRT or EC in this trial) are more effective when delivered alongside behavioural support [22]. Stop smoking specialists are trained in delivering 16 evidence-based behaviour change techniques (BCTs) as part of intensive weekly behavioural support [23]. However, intensive behavioural interventions, such as weekly support, would be unrealistic in busy NHS primary care dental settings. Additionally, the evidence suggests that the dose–response curve is shallow for behavioural support when combined with pharmacotherapy, i.e. less intensive interventions are not greatly inferior to more intensive ones. Hence, in keeping with the pragmatic design of this trial, we have chosen to use a single-visit behavioural smoking cessation intervention (Table 1). This intervention will include the 5 most evidence-based and appropriate BCTs that have been used in the intensive weekly behavioural support [24].

**Table 1:** Single-visit smoking cessation behavioural intervention delivered in ENHANCE-D

Behaviour change technique	How delivered?
Assessing current and past smoking behaviour	Confirm current and past smoking behaviour by discussion with participant. This information is likely to already be known from the medical history but should be revisited
Providing information on consequences of smoking and smoking cessation	Inform participants that smoking can lead to: • Tooth staining • Gum disease • Tooth loss • In severe cases, mouth cancer Stopping smoking to help reduce the chances of these or make dental treatments work better
Assessing current readiness and ability to quit	Explore readiness to quit by discussion
Facilitating goal setting	Set goals such as a quit plan or cutting down to quit (while using NRT/ECs)
Offering appropriate written materials	Provide the Cancer Research UK ‘You can be Smoke Free’ leaflet. Available at: https://publications.cancerresearchuk.org/publication/you-can-be-smoke-free Any local smoking cessation leaflets as per usual care Medication package inserts will be included with the NRT or ECs as appropriate

In this trial, the intervention is a single-visit behavioural support intervention along with the offer of NRT or an EC starter kit. The products offered are available over the counter in the UK without prescription, and therefore the participant can choose to stop or start using the NRT or EC at any time.

### Trial outcomes

#### Primary outcome

Biochemically verified smoking abstinence at 6 months from baseline visit (an established measure of long-term smoking abstinence) using a carbon monoxide monitor. An expired air carbon monoxide (eCO) reading of 10 parts per million (ppm) or above signifies smoking tobacco.

### Key secondary outcome

Periodontitis sub-study: Percentage of periodontal sites at 6 months with PPDs (probing pocket depths) ≥ 5 mm (a standard outcome measure of periodontal health for periodontal studies; indicates a site that is ‘diseased’ with an increased risk of subsequent tooth loss [25]).

### Other secondary outcomes

The following outcomes will be measured in all the participants:

- Adverse events (shortness of breath, cough, phlegm, nausea, throat/mouth irritation, sleep disturbance, headache, mouth dryness, dizziness/feeling faint)
- Continuous biochemically verified smoking abstinence at 12 months (a long-term measure of smoking abstinence)
- Fagerstrom Test for Nicotine Dependence (FTND) [26], a measure of the degree of dependence among smokers coming to a smoking cessation clinic
- Mood and Physical Symptoms Scale (MPSS) [27]
- Oral Health Quality of Life Assessment (OHQoL-UK) [28]
- Expired air carbon monoxide (eCO; for patients who attend follow-up visits)
- Number of teeth
- Costs to the NHS and participants
- Incremental cost per smoking abstinence
- Net monetary benefits based on participants’ willingness-to-pay for the interventions and associated outcomes
- Incremental net benefit
- Qualitative evaluation

The following outcomes will be measured in the periodontitis sub-study only:

- GI (Gingival Index [Lobene Modified Gingival Index [29]]; a measure of gingival health)
- PI (Plaque Index [Silness and Loe plaque index [30]]; a measure of oral hygiene)
- REC (Gingival recession; a measure of loss of gingival tissue/attachment)
- CAL (Clinical Attachment Loss; a measure of current and previous periodontal disease exposure; derived from PPD and REC)
- BOP (Bleeding on Probing; a measure of gingival health)
- CODS (Clinical Oral Dryness Score [31])
- PESA (Periodontal Epithelial Surface Area; a novel method of measuring periodontal health; derived from PPD data [32])
- PISA (Periodontal Inflamed Surface Area; a novel method of measuring periodontal health; derived from PPD and BOP data [32])
- Biological samples (subgingival dental plaque and buccal brush biopsy) for exploratory analyses
- A sub-study aims to assess socioeconomic inequalities in trial participation.

### Blinding

Prior to randomisation, participants will be blinded to the purpose of the trial and will not be aware of the treatment interventions being offered. The trial will be described in terms of different packages of advice that the dental team can offer rather than describing the interventions in detail (VBA, NRT, EC). Prior to randomisation, the participants will also be unaware that the specific aim of the trial is smoking cessation.

This is a common approach in research studies where some of the interventions are light touch, such as the brief intervention (control group) in this trial. This is done because by explaining the interventions in the PIS, the dental professional has effectively delivered the control brief intervention. Similar approaches have been used in a range of studies [33–35], including a recent smoking cessation study [36]. The PIS will detail that if any ‘medication or other devices’ are offered by the dental professional, the participant can decide not to use these, but if they do, any side effects, benefits, and risks will be discussed by the dental professional to inform the participant’s decision. Participants will receive a randomisation group-specific PIS following randomisation.

For the primary outcome (biochemically verified smoking abstinence at 6 months), assessors will not be blinded to participant allocation or smoking status, but an objective measure will be taken to verify self-reported tobacco abstinence (eCO). For the key secondary outcome (percentage of periodontal sites at 6 months with PPD ≥ 5 mm), the outcome assessors will be blinded to participant allocation and smoking status. Participants will be asked not to disclose their smoking status or methods of smoking cessation to the blinded outcome assessor. There are three trial statisticians: two senior statisticians and a trial statistician. One senior statistician will remain blind to treatment allocation until the end of trial database lock. The remaining two statisticians will not be blinded to treatment allocation.

### Data collection

Participant data will be entered by the PI and their delegated nominees into the secure, trial-specific Clinical Data Management System (CDMS):Sealed Envelope’s Red Pill database.

The storage of personal data will be treated as confidential and stored in accordance with the Data Protection Act 2018 and General Data Protection Regulation. Access is limited to authorised users at site and Newcastle University.

Users are assigned role-based permissions specific to their site and study role. Patient identification will be through a unique participant ID, allocated at screening. These IDs will specify hub, site, and individual patient and will be provided sequentially at each site.

Participants will be completing questionnaires for this trial on paper forms at baseline and 6 months. These will be transcribed by the site staff onto the database, and the paper originals will remain at the site. Validations will be built into the Red Pill database, while additional manual validations will also be made to promote data quality. Data management processes are described in the data management plan.

All participants will be given a voucher to the value of £20 for completing the baseline visit. To encourage retention, further £20 vouchers will be given when they complete the 6- and 12-month follow-ups. For those participants who fail to attend the 6-month or 12-month follow-up visit, a member of the site research team will attempt to contact them via telephone or SMS message and establish self-reported smoking status and any adverse events. The option of remote collection through posting out the carbon monoxide monitor will be made available for those who cannot attend in person.

The study data collected are outlined in the Schedule of Events (Table 2). Details of each study measure are given in Additional file 1.

**Table 2:** Schedule of events

**Procedures**	**Screening**	***Baseline*** ***(Part 1)***	Day 7* (+3 days)	**Day 21**^**$**^ (± 2 days)	**Day 28** (+5 days)	**6 months**^**@**^	**12 months**^**@**^
Informed consent	**X**						
BPE (if not done in previous 3 months)	**X**						
Eligibility assessment	**X**						
Randomisation		***X*** *No more than 3 days before intervention & ordering product*					
Demographics		***X***					
Medical/smoking history (# self-reported smoking status, including type and amount)		***X***				**#**	**#**
Number of teeth (this can be taken from recent BPE)		***X***				**X**	
Expired air carbon monoxide						**X**	**X**
Fagerstrom Test for Nicotine Dependence (FTND)		***X***				**X**	
Mood and Physical Symptoms Scale (MPSS)		***X***				**X**	
Oral Health Quality of Life Assessment (OHQoL-UK)		***X***				**X**	
Health service utilisation questionnaire		***X***				**X**	
AEs and SAEs will be recorded					**X**	**X**	**X**
Concomitant medications (used for AEs)					**X**	**X**	**X**
Behavioural intervention		X*					
Ordering of trial products		X* *Within 48 hours of delivering behavioural intervention*		**X**^**$**^			
Confirmation of receipt of trial products			X*		**X**^**$**^		
Contingent valuation study						**X**	
Gift Voucher		***X***				**X**	**X**
***The following assessments are for the Periodontitis sub-study ONLY—collected by the regional blinded hub assessors.***							
PPD (Probing Pocket Depth)		***X***^***P***^				**X**^Q^	
GI (Gingival Index [Lobene Modified Gingival Index])		***X***^***P***^				**X**^**Q**^	
PI (Plaque Index [Silness and Loe plaque index])		***X***^***P***^				**X**^Q^	
Gingival recession		***X***^***P***^				**X**^Q^	
BOP (Bleeding on Probing)		***X***^***P***^				**X**^Q^	
CODS- Clinical Oral Dryness Score		***X***^***P***^				**X**^Q^	
Collection of periodontal therapy details						**X**^Q^	
***Periodontitis sub-study – Biological Sample collection***							
Sub gingival dental plaque		***X***^***P***^				**X**^Q^	
Buccal brush biopsy		***X***^***P***^				**X**^Q^	
Unstimulated saliva (exploratory data collection in selected sites only)		***X***^***P***^				**X**^Q^	
Calculus sample (exploratory data collection in selected sites only)		***X***^***P***^				**X**^Q^	

*= if randomised to NRT or EC $=If randomised to NRT group only

^@^* =The follow-up visits are scheduled around the usual 6 monthly recall visits that would be recommended for someone with a risk factor such as tobacco smoking. Ideally this should fall 6 and 12 months from the baseline visit, but no specific visit timing parameters are defined*

^P^*=Baseline: before (maximum of 28 days prior) or on the day of delivery of the intervention*

^Q^*= on the same day as, or a maximum of 28 days after, the 6-month follow-up visit conducted by the primary care team*

### Periodontal sub-study

Additional assessments will be conducted at baseline and 6-month visits for the participants in the periodontal sub-study. These will be conducted by the regional hub assessors, who are blinded to the participant’s trial randomisation group and smoking status. Training sessions were held for the examiners (dentist, dental therapist or dental hygienist) and dental nurses from each hub before the first patient recruitment, in the form of an in-person training event in the Newcastle Dental Clinical Research Facility. A range of examiner alignment activities were undertaken. Inter-examiner agreement was assessed against a gold standard, at the mouth level, to ensure the reliability of the inputs to the statistical models for the final analyses. Repeat measures were used to assess intra-examiner reliability. Live data were presented during the sessions, and additional training and repeat exercises completed where necessary. If new examiners join the study, they will be required to complete the examiner alignment activities individually.

The assessments that will be conducted by the hub assessors at baseline and 6 months on participants in the periodontal sub-study are listed in the schedule of events (Table 2).

Sub-study participants will receive an additional £20 voucher on completion of baseline and 6-month assessments. Assessors from the hub will visit the participant’s dental practice to perform the assessments, or in some cases the participant may travel to the hub, in which case they will receive a further additional incentive in the form of a £20 gift voucher to cover any travel expenses.

### Sample size

**Fig. 1:**
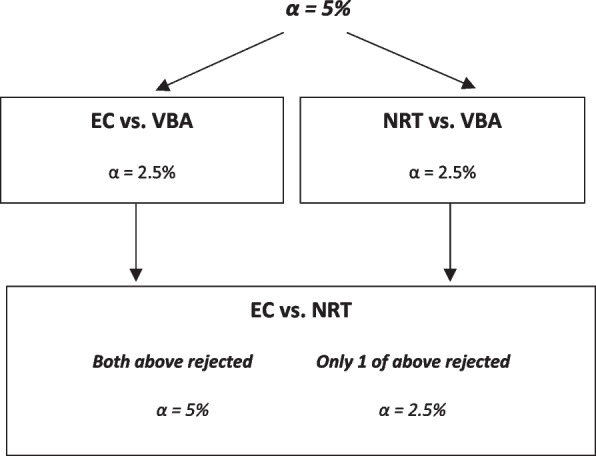
Bonferroni-based gate keeping method

The sample size calculation was based on a test of the superiority of either NRT or EC compared to VBA, for the primary outcome, according to the Bonferroni-based gatekeeping method (Fig. 1) [37]. If at least one of the two hypothesis tests for NRT or EC compared to VBA is rejected, we will test the superiority of EC compared to NRT.

Assuming a rate of biochemically verified smoking abstinence at 6 months for VBA of 6% [38, 39] with a minimal clinically important difference (MCID) of 7% [13, 40], 85% power and type 1 error rate of 2.5%, a total of 1215 participants (VBA= 243; NRT/EC = 486) would be required to perform a one-sided test for each hypothesis (NRT vs VBA, EC vs VBA). If both null hypotheses are rejected, we will carry forward a type 1 error rate of 5%, and based on an NRT rate of 11%, we can detect a difference of 7% with 93% power and 6% with 85% power (one-sided test EC vs NRT). If only one null hypothesis is rejected, we will carry forward a type 1 error rate of 2.5% with which we can detect a difference of 7% with 87% power. Note that this is not inflated for attrition in line with standard research practice in this field [41].

Further details of the sample size calculations for the trial are included in Additional file 1.

### Statistical analysis

#### General analysis principles

Analyses will be conducted for available data according to the ITT principle. Results will be presented as the treatment effect with the *p*-values extracted directly from the model. All comparisons will also be presented with 95% confidence intervals [42]. For the primary outcome, statistical significance will be at the level determined by the Bonferroni-based gatekeeping method (Fig. 1). For all other outcomes, we will present the results of the unpowered comparisons with 2-sided *p*-values, where *p*-values < 0.05 will be considered statistically significant.

#### Analysis of the primary outcome

The primary outcome of biochemically verified smoking abstinence at 6 months will be analysed using mixed-effects logistic regression adjusted for the stratification factors, research hub/region (random effect), and baseline periodontal status (fixed effect). Average marginal effects between randomisation groups will be calculated from each model to estimate the risk differences at 6 months (EC-VBA, NRT-VBA, EC-NRT). Any participants with missing smoking abstinence data will be regarded as still smoking [41].

A tipping point analysis will be conducted to assess the sensitivity of the assumption that if abstinence is not verified, the participant is still smoking. Details will be given in the pre-specified Statistical Analysis Plan (SAP).

#### Analysis of the key secondary outcome (periodontitis sub-study)

The key secondary outcome of the percentage of periodontal sites with PPD ≥ 5 mm at 6 months, for participants included in the periodontitis sub-study, will be analysed using a linear mixed-effects model adjusted for the primary outcome and for baseline percentage of sites with PPD ≥ 5 mm.

Missing data mechanisms will be considered, and sensitivity analyses may be carried out on an imputed dataset— these will be pre-specified in the SAP.

#### Analysis of all other secondary outcomes

The binary secondary outcome measures, such as continuous biochemically verified smoking abstinence at 12 months, will be analysed using a mixed-effects logistic regression model. Numerical secondary outcome measures, such as Oral Health Quality of Life Assessment score, will be analysed using a linear mixed-effects regression model. All models will be adjusted as for the primary outcome, with the addition of the adjustment for the baseline of that outcome if available. Details will be given in the pre-specified Statistical Analysis Plan (SAP).

#### Interim analyses

There are no formal interim analyses planned in this trial.

#### Additional analyses

An analysis investigating socioeconomic inequalities in participation in the ENHANCE-D trial will be carried out as part of a related sub-study.

### Economic evaluation

The health economic component will include an economic evaluation in the form of a cost-effectiveness analysis (CEA) and a cost–benefit analysis (CBA). The economic evaluation will be conducted according to best practice guidelines [43].

The CEA will use the trial primary outcome (smoking abstinence) as the outcome measure. Alongside the CEA, a CBA will also be conducted using a measure of willingness-to-pay (WTP) for the interventions and primary outcome (smoking abstinence at 6 months). WTP values for the CBA will be elicited via a contingent valuation study (CVS), in which preferences for each intervention and the primary outcome are obtained. The CBA will be administered at 6 months via an online survey, either completed at the practice visit or at home by participants. The survey will take the form of a payment card [44]. The values used in the CVS were informed by the relevant literature, trial team and PPI representatives.

Data will be collected within the trial and used to calculate costs and estimate outcomes for the CEA. The interventions will be micro-costed, and bespoke participant questionnaires (Healthcare Utilisation Questionnaire) and eCRF will capture data on medications (only medications related to smoking cessation and managing any symptoms associated with smoking cessation will be considered), dental service use, and other subsequent healthcare service use. Participant questionnaires, created using input from the PPI representatives, will be administered at baseline and 6 months post-randomisation to capture service use. Unit costs for these services will be obtained from routine data sources and trial-specific estimates [45]. The analysis will be conducted from an NHS and personal and social services perspective. Unit costs will be combined with service use to estimate costs per participant in each randomisation group of the trial. Sensitivity analyses will estimate the costs incurred by participants in the trial. Participant costs will be collected as part of the participant questionnaires.

For the CEA, the results will be presented as point estimates of mean incremental costs and effects. Regression analysis will be applied to costs and effects to estimate adjusted point estimates of incremental costs, effects and cost-effectiveness [46]. Stochastic analyses (plots of cost and effects and cost-effectiveness acceptability curves) using bootstrapping techniques will be conducted to explore uncertainty in the estimate of cost-effectiveness [47–49]. The results of the CBA will be presented as incremental costs and effects (effectiveness will be estimated by the mean WTP for smoking cessation multiplied by the primary outcome) and incremental net benefits, where net benefits = incremental effects – incremental costs. The results of the CBA will also be used to estimate the net benefits of each intervention which will be determined by mean WTP of the intervention – mean costs of the intervention.

Deterministic sensitivity analyses will be performed to explore key uncertainties. Where appropriate, these analyses will be combined with a stochastic analysis with the results presented as described above. Missing data is expected from the trial; methods of imputation to address missing data will be determined once the full dataset is available [50]. Both the CEA and CBA will include an assessment of the distribution of outcomes according to socioeconomic status to explore any possible impacts of the intervention on inequalities.

An analysis of the periodontitis sub-study will use the same methods as described above for the CEA. This analysis will be largely exploratory as it is expected that the sub-study sample size will be insufficient for robust conclusions to be drawn on cost-effectiveness.

### Qualitative evaluation

A qualitative study within the main trial will explore participants’ experiences of receiving the study interventions whilst incorporating the views of dental professionals and NHS commissioners or service managers. The latter groups may identify additional facilitators or barriers to these interventions being adopted in NHS primary dental care. The study will adopt Normalisation Process Theory [51] (NPT) to guide data interpretation. NPT comprises four constructs: Coherence, Cognitive Participation, Collective Action, and Reflexive Monitoring [52]. This qualitative study may involve up to 32 patients, 16 dental professionals, and 5 NHS commissioners. These indicative numbers are based upon predictions linked to purposive maximum variation sampling, including the variables of sex, age, UK region, the dental practice where the intervention is delivered, and patients representing all trial randomisation groups. Based upon the authors’ collective experiences of conducting qualitative research in dental settings, it is anticipated that theoretical saturation is likely to be achieved by the numbers stated above. The sample will include a range of dental team members involved with delivering the trial interventions and working under different NHS dental contractual regulations. Selected participants recruited via purposive maximum variation sampling will be invited to participate in this qualitative study at two time points: at approximately 1 month following initial receipt of their allocated intervention and after their 6-month appointment. Dental professionals will be invited to participate on the same basis, but NHS commissioners/service managers will be interviewed once at approximately 9–12 months following the start of trial recruitment.

Qualitative data collection methods will offer a choice of face-to-face or online semi-structured interviews, telephone interviews, or focus group meetings. All interviews and focus groups will be audio-recorded, following the structure outlined in topic guides customised to each participant group based upon the research team’s prior experience in this area of study (see Additional File 3). Topic guides will be revised during the data collection process if required. Interviews and focus groups will be led and analysed initially by a single experienced qualitative researcher. Sampling sufficiency will be monitored on an ongoing basis throughout the data collection period, and this process will similarly consider the richness and diversity of the data obtained from the participant sub-groups involved. Recordings will be transcribed verbatim by a professional transcription company. Data will be analysed thematically and managed with the support of NVivo qualitative data software (Lumivero, Version 13, released March 2020). Regular meetings involving three members of the research team will aim to achieve consensus during coding, resulting in final, agreed themes for subsequent dissemination. The research team will follow guidance published in ‘Standards for reporting qualitative research: a synthesis of recommendations’ (SRQR).

### Safety reporting

All interventions in ENHANCE-D are licensed products with well-established safety profiles. The side effects for NRT products are extensively documented; therefore, several adverse events of interest relating to ECs will be collected at the follow-up timepoints (see schedule of events). The pre-specified AEs are shortness of breath, cough, phlegm, nausea, throat/mouth irritation, sleep disturbance, headache, mouth dryness, and dizziness. There is also an option for investigators to record other unspecified adverse events. Serious adverse reactions (defined as any untoward medical occurrence that results in death, is life-threatening, requires inpatient hospitalisation or prolongation of existing hospitalisation, results in a significant disability/incapacity or consists of a congenital anomaly or birth defect) which are judged to be related to the use of NRT/EC, will also be reported (prior to protocol version 5 [16SEP2024] any event meeting the criteria of an SAE was reported).

## Trial oversight committees and monitoring

The Trial Management Group (TMG) will be responsible for the day-to-day running of the trial and will comprise the CI, co-investigators, members of NCTU, statistician(s), sponsor, and, as required, other members of the co-applicant team. The TMG will monitor all aspects of trial conduct and progress, protocol adherence and take appropriate action to safeguard participants and the quality of the trial itself. The TMG will centrally monitor recruitment, data collection rates and protocol compliance. The TMG will meet every 4–6 weeks. The Trials Unit will perform onsite monitoring once during the trial in at least 50% of sites to ensure GCP compliance and protocol adherence. An Independent Data Monitoring Committee (IDMC) and Trial Steering Committee (TSC) will be established as per NIHR guidance [53], composed of independent experts in the field (clinicians and statisticians) and patient and public representatives (see Additional file 1 for full composition). The role of each committee is defined in their Charter. No formal interim analysis is planned. The IDMC will make recommendations to the TSC as to whether there are any ethical or safety issues that may necessitate changes to the trial.

## Patient, public, and stakeholder involvement

A co-production approach was employed in the design of this trial utilising the expertise of patients, members of the public, dental professionals and other key stakeholders. Following several small group sessions (e.g. a condition-specific patient panel) we held a ‘Co-Production Grand Session’ with over 25 members from a wide range of stakeholder backgrounds. The TMG includes a lay co-applicant and the TSC has two PPI representatives.

## Regulatory, ethics approval, and consent to participate

The trial is categorised as a type A trial and was submitted through the combined review process. Favourable ethical opinion was received from the North East – Tyne & Wear South Research Ethics Committee (reference: 22/NE/0040). Written informed consent will be obtained from all participants. The trial received REC, MHRA & HRA approval on 05 April 2022. All substantial protocol amendments will receive approval by sponsor, REC, HRA, and MHRA (where applicable); these will then be distributed to participating sites, clinical research networks, and investigators. The trial registries will be updated accordingly.

## Dissemination plans

To communicate with academics and medical professionals, the intention is to publish several scientific papers in peer-reviewed publications and to present lectures and posters at national and international academic conferences. The final report to the funder will be published in the NIHR Health Technology Assessment (HTA) journal. An easily accessible study summary will be prepared for wider policy and practice audiences. We plan to conduct a national roadshow, focused on our hub locations, to share the research findings with the wider dental community.

## Discussion

This is planned to be one of the largest interventional dental trials conducted in the UK. It has been designed to be highly pragmatic in its nature from the outset—the PRECIS-2 tool [54] was used during the design stage to ensure consistency in this approach (https://www.precis-2.org/Trials/Details/521).

There are several aspects of the study design worthy of further explanation. The definition of smoking status is based on the patient’s self-identification as a ‘regular smoker’. There is no threshold for the number of cigarettes smoked per day as often used in other studies. This approach was developed during the co-production stage of trial design following the external pilot study where patients often struggled to define their smoking habit in this rigid way. In real life, there is no threshold for providing smoking cessation interventions; all smokers are offered support, so following the pragmatic nature of the trial, we adopted a similar approach.

Participants are blinded to the purpose of the trial and not aware of the other trial interventions. There were several reasons for this approach. Firstly, to reduce cross-over between the randomisation groups. During the external pilot study [15], 20% of the control group reported sourcing their own EC, and it was felt a factor in this was them knowing that other participants in the trial were being offered an EC. The second reason was around the VBA intervention control group, a 30s intervention. If we had explained the intervention in the participant information, then we would have effectively delivered the intervention. This approach is commonly taken in studies of brief interventions across a range of disciplines [33–36]. Several other aspects of the trial were designed to support this by ensuring there was not excessive focus on smoking, which could have unintentionally enhanced the VBA intervention. These included: the smoking interventions being incorporated into a wider package of advice covering toothbrushing, fluoride, diet, alcohol, and smoking (as would be normal practice in usual care); eCO not being measured at baseline but only at follow-up; sites being trained in how to introduce and discuss the study without excessive focus on smoking, instead describing the study as being about evaluating different packages of advice from the dental team.

A central pharmacy was used for the supply of the NRT and EC products. We had considered if each dental practice site could have its own supply of intervention products as this would have been ideal for the provision of products to the participants immediately after their behavioural interventions. However, this was deemed to be impractical due to the substantial number of flavour and strength options that would need to be kept in stock across 56 sites. We also considered using local community pharmacies, but this was also deemed impractical due to them not routinely stocking EC products. Using a central pharmacy allowed us to standardise the intervention offer, and this approach could be used as an efficient model of provision of these interventions if rolled out after the trial.

We anticipate one of the biggest challenges in this trial will be conducting it in primary dental care. These settings are busy with large pressures on clinical time, and they often have little or no research experience. In keeping with the pragmatic nature of the trial, we worked with primary dental care team members during the co-production process to ensure the trial was as practical as possible to deliver. For example, recruitment strategies were very flexible to ensure all possible circumstances were accommodated, and follow-up periods were designed around usual recall periods. Our qualitative evaluation will identify facilitators or barriers to the study interventions being adopted in NHS primary dental care.

### Trial status

The current protocol version is V5.0 dated 16SEP2024. Participant recruitment began on 15/07/2022 and is expected to be completed by 31/03/2025.

## Supplementary Information

Additional file 1: Supplementary material

Additional file 2: SPIRIT checklist

Additional file 3. Topic guides

## Data Availability

Until publication of the trial results, access to the full-blinded dataset will be limited to the Trial Management Group and to authors of the publication. Explicit consent is obtained via the informed consent form from each trial participant to allow data sharing to occur. De-identified data from this trial may be available to the scientific community subject to appropriate ethical approval. Requests for data should be directed to the lead author/Chief Investigator and Clinical Trials Unit.
