# Supplementary material for: ENHANCE-D: protocol for a pragmatic, 3-arm, randomised controlled trial comparing the impact of enhanced smoking cessation interventions to very brief advice for adult smokers in dental care settings
Source: Trials. 2025 Jul 28;26:261. doi: 10.1186/s13063-025-08954-z (PMC12305899; doi:10.1186/s13063-025-08954-z)
Supplement: Supplementary file 1 — Additional file 1: Supplementary material [file 13063_2025_8954_MOESM1_ESM.docx]

**ENHANCE-D: Protocol for a pragmatic, 3-arm, randomised controlled trial comparing the impact of enhanced smoking cessation interventions to very brief advice for adult smokers in dental care settings.**

**Additional file 1**

Richard Holliday^1,2^, Nina Wilson^3^, Vicky Ryan^3^, Chrissie Butcher^4^, Tara Homer^3^, Philippa Watts^4^, Dorcas Kareithi^3^, Paul Blaylock^1^, Laura Ternent^3^, Roland Finch^5^, Susan M. Bissett^1^, Adam Todd^6^, Helen Hancock^3,4^, Fiona Ellwood^7^, David I Conway^8^, Nicholas S. Jakubovics^1^, Ralf Kist^1^. Richard D. Holmes^1^ Linda Bauld^9^, Philip M. Preshaw^1,10^, Elaine McColl^3^

Contents

[1. Internal Pilot 1](#_Toc184287239)

[2. E-cigarette (EC) starter kit 2](#_Toc184287240)

[3. Data Collection 3](#_Toc184287241)

[4. Changes to the original sample size 6](#_Toc184287242)

[5. Oversight Committee composition 7](#_Toc184287243)

[6. Additional file 1 references 8](#_Toc184287244)

## 1. Internal Pilot

ENHANCE-D had an internal pilot designed to assess a range of parameters. The duration was 8 months, from the start of the recruitment period, based on when we predicted to have recruited 25% (n=365) of the participants and have all sites open. The progression criteria used are detailed below:

| **Progression criteria** | **Red % (n)** | **Amber % (n)** | **Green %(n)** |
| --- | --- | --- | --- |
| Total number of participants recruited | <50% (182)  Discuss with oversight groups. Consider feasibility of continuing to main trial, any possible mitigations and make proposals to discuss with Funder. | 50-99% (183-364)  Discuss with oversight groups. Propose a recovery plan to agree with Funder. | ≥100% (365)  Proceed with main trial |
| Number of sites opened as % of expected | <50% | 50-99% | 100% |
| Proportion of recruited participants who are in the periodontitis subgroup | This information will be used to determine if any additional recruitment steps need to be taken to ensure recruitment to the periodontitis subgroup. | | |

Recruitment and trial procedures continued during the decision-making process.

## 2. E-cigarette (EC) starter kit

Customised e-cigarette starter kit to include:

- Aspire PockeX e-cigarette
- Coil replacement pack
- 3-pin plug
- 10 x standard 10ml e-liquids

Choice of four e-liquid bundles:

* If an e-liquid flavour becomes unavailable, it will be substituted for a similar flavour.

| A (Tobacco focus)* | 4 x Tobacco 1.8%  3 x Tobacco 1.2%  1 x Menthol 1.8%  1 x Fruit A (e.g. Raspberry) 1.8%  1 x Fruit B (e.g. Mango) 1.8% |
| --- | --- |
| B (Menthol focus)* | 4 x Menthol 1.8%  3 x Menthol 1.2%  1 x Fruit A (e.g. Raspberry) 1.8%  1 x Fruit B (e.g. Mango) 1.8%  1 x Tobacco 1.8% |
| C (Full mix)* | 2 x Menthol 1.8%  1 x Menthol 1.2%  1 x Fruit A (e.g. Raspberry) 1.8%  1 x Fruit A (e.g. Raspberry) 1.2%  1 x Fruit B (e.g. Mango) 1.8%  1 x Fruit B (e.g. Mango) 1.2%  1 x Tobacco 1.2%  2 x Tobacco 1.8% |
| D (Non-tobacco mix)* | 2 x Menthol 1.8%  1 x Menthol 1.2%  2 x Fruit A (e.g. Raspberry 1.8%  1 x Fruit A (e.g. Raspberry 1.2%  2 x Fruit B (e.g. Mango) 1.8%  2 x Fruit B (e.g. Mango) 1.2% |

## 3. Data Collection

The following data will be collected from all participants at baseline:

- **Demographics**

1. Age
2. Sex
3. Ethnicity
4. Home postcode
5. Highest level of educational level obtained
6. Job title, or reason for not working
7. Receipt of low-income benefits

- **Medical/ Smoking History**
  - - - Type of tobacco used (factory made cigarettes, hand rolled cigarettes, cigars, pipes)
      - Current amount of tobacco/day (cigarettes/day or weight of tobacco/day)
      - Total pack years
      - Any previous quit attempts. Date of last attempt
      - Any current or previous use of NRT and EC
    - **Number of teeth**

The number of teeth present will be recorded from the latest dental charting on the dental records (i.e. no additional examination will be conducted).

- - - **Fagerstrom Test for Nicotine Dependence (FTND)**

This tool assesses the degree of dependence among smokers coming to a smoking cessation clinic and is extensively used in tobacco research [1]. It consists of a set of six questions, giving a score of 0-10 with higher scores representing heavier smokers.

- **Mood and Physical Symptoms Scale (MPSS)**

This 12-item questionnaire assesses cigarette withdrawal symptoms. Each item gives a maximum score of 5 giving a total maximum score of 60. Ten of the items have a minimum score of 1 and two have a minimum score of 0, giving an overall minimum score of 10. A higher score indicates worse withdrawal symptoms. It has been used for over 30 years with its psychometric properties being assessed by West and Hajek, 2004. To assess the effect of abstinence you can calculate the change from baseline (just prior to stopping smoking) to the post-abstinence follow-up point for items 1. to 7. and 10. to 12., and take the raw scores for items 8 and 9 [2]. To compare abstinence symptoms under two or more conditions (i.e. the two arms of this trial) these scores can be compared or instead the post-abstinence ratings compared using the baseline ratings as covariates (i.e. instead of subtracting them). This method gives slightly more power to detect differences. The ratings will be analysed individually but also totalled together to give a composite score (MPPS Total).

- **Oral Health Quality of Life Assessment (OHQoL-UK)**

The OHQoL-UK questionnaire [3] will give a measurement of oral health related quality of life at two points in the trial (baseline and 6-months). The 16 items allow responses in either a positive or negative (bidirectional) manner to a series of statements about the effect of oral health on specific aspects of respondents’ daily lives. The responses range from “very bad” (score 1) to “very good” (score 5). Responses are then summed to give a total score out of 80, or can also be summed within three sub-domains (physical, social and psychological) as described by McGrath and Bedi [4]. The lower the score the poorer the OHRQoL.

- **Health service utilisation questionnaire**

The following will be collected from all participants 6 months after randomisation:

**All participants:**

- **Self-reported smoking status**

The smoking status including type and amount will be recorded.

- **Expired air Carbon Monoxide**

In accordance with national guidelines [5], the trial will measure eCO on all participants at 6-months and 12-months. A carbon monoxide monitor will be used.

- **Number of teeth**
- **Fagerstrom Test for Nicotine Dependence (FTND)**
- **Mood and Physical Symptoms Scale (MPSS)**
- **Oral Health Quality of Life Assessment (OHQoL-UK)**
- **Health service utilisation questionnaire**
- **Adverse events and concomitant medication for AEs.**

***Assessments for the periodontitis sub group only –*** *conducted by the regional blinded hub assessors:*

Assessors from the hub will visit the participant’s dental practice to perform the assessments, or in some cases the participant may travel to the hub, in which case they will receive an additional incentive in the form of a £20 gift voucher to cover any travel expenses.

- **Clinical Oral Dryness Score (CODS)**

Oral dryness (xerostomia) will be measured using a 10 point scale as described by Osailan et al [6]. A score of 1 is assigned for each of the following:

1) mirror sticks to buccal mucosa;

2) mirror sticks to tongue;

3) frothy saliva;

4) no saliva pooling in floor of mouth;

5) tongue shows loss of papillae;

6) altered/smooth gingival architecture;

7) glassy appearance of other oral mucosa, especially palate;

8) tongue lobulated/fissured;

9) active or recently restored (last 6 months) cervical caries (>2 teeth); and

10) debris on palate (excluding under dentures).

- **Gingival Index (GI [Lobene Modified Gingival Index])**

A full mouth gingival index will be recorded based on the Lobene Modified Gingival Index [7] (MGI) rated on a scale of 0 to 4 (recorded at 6 sites per tooth):

| 0 | Absence of inflammation |
| --- | --- |
| 1 | Mild inflammation; slight change in colour, little change in texture of any portion of but not the entire margin or papillary gingival unit |
| 2 | Mild inflammation; but involving entire margin or papillary unit |
| 3 | Moderate inflammation; glazing, redness, oedema and/or hypertrophy of margin or papillary unit |
| 4 | Severe inflammation; marked redness, oedema and/or hypertrophy of marginal or papillary gingival unit, spontaneous bleeding, congestion, or ulceration] |

- **Plaque Index (PIn [Silness and Loe plaque index])**

The plaque index of Silness and Loe [8] will be employed to measure plaque (without disclosing) at 6 sites per tooth.

Scores will be assigned as follows:

| 0 | No plaque |
| --- | --- |
| 1 | A thin film of plaque at the gingival margin which may be seen only after running the probe along the tooth surface |
| 2 | Moderate accumulation of plaque deposits which can be seen with the naked eye |
| 3 | Extensive accumulation of plaque deposits |

- **Gingival recession (used to calculate CAL outcome measure)**

Gingival recession will be recorded to the nearest millimetre using a manual UNC-15 periodontal probe. Gingival recession is the distance from the free gingival margin to the cemento-enamel junction. Gingival recession will be indicated as a positive number. If gingival overgrowth is present then 0 will be recorded i.e. there will be no negative numbers recorded as recession.

- **Pocket Probing Depths (PPD)**

A trained and calibrated hygienist, blinded to group allocation, will collect the PPDs using a manual UNC-15 periodontal probe to record the probing depths to the nearest millimetre. Probing depth is the distance from the probe tip (assumed to be at the base of the pocket) to the free gingival margin. Recorded at 6 sites per tooth.

- **Bleeding on Probing (BOP)**

Following probing, each site will be assessed for bleeding on probing, if bleeding occurs within 10s of probing, a score of 1 will be assigned for the site, otherwise, a score of 0 will be assigned. Recorded at 6 sites per tooth.

- **Periodontal Epithelial Surface Area (PESA)**

PESA quantifies the root surface area affected by attachment loss. It is calculated from the PPDs using the technique described by Nesse 2008.[9]

- **Periodontal Inflamed Surface Area (PISA)**

PISA quantified the surface area of inflamed periodontal tissue. It is calculated from the PPDs and BOP data using the technique described by Nesse 2008.[9]

- **Biological sample collection**

Subgingival dental plaque will be collected from 10 periodontal pockets (ppd ≥ 5mm) using sterile paper points and pooled. If less than 10 pockets are available, then as many as possible will be used. The same sites will be used at both collection visits. If a site is not available at the 6-month visit, due to tooth extraction, no additional sites will be added. Samples will be stored dry and shipped to Newcastle University where they will be stored at -80°C until analysis.

Oral epithelial cell samples will be collected using buccal brush biopsies. Cells will be stored in RNA protection reagent and shipped to the laboratory for RNA extraction. The presence of oral potentially malignant disorders (OPMDs) at the site where the buccal brush biopsies are collected will be recorded.

- **Exploratory biological sample collection**

Unstimulated saliva will be collected in the periodontitis subgroup at selected hub locations. This will be collected by drooling into a collection vessel until approximately 5ml has been collected (estimated to take 5-10 minutes). Participants will be asked if they have eaten, drank, taking medication or performing oral hygiene tasks in the hour prior to collection. Samples will be frozen on return to the hub base.

Calculus samples will be collected in the periodontitis subgroup at selected hub locations. Samples will be frozen on return to the hub base.

## 4. Changes to the original sample size

The original target sample size for the study was 1460 participants. This calculation was based on a test of the superiority of either EC or NRT compared to VBA for the primary outcome (biochemically verified smoking abstinence) and the same for the key secondary outcome (percentage of periodontal sites at 6 months with PPD ≥ 5mm for patients with periodontitis), according to the Bonferroni-based gatekeeping method [10]. However, recruitment to the periodontitis sub-study was slower than initially anticipated and it was decided to no longer power this outcome, reducing the sample size to 1215. This reduction in the sample size was the result of no longer needing to split the type 1 error rate between the primary outcome and the key secondary outcome.

It was estimated that 31% of the original 1460 participants (n=455) would be eligible for the periodontitis sub-study. At screening, all patients are assessed for eligibility for the periodontitis sub-study, but not all those eligible will be enrolled in the sub-study. One of the original seven research hubs did not open to the periodontitis sub-study. Two opened but never recruited, leaving four who recruited to the periodontitis sub-study. Additional research regions were added during the study to boost recruitment to the main study; these did not recruit to the periodontitis sub-study with all their participants entering the main trial.

Using an estimate of the standard deviation of percentage of periodontal sites at 6 months with PPD ≥ 5mm of 10.1% [11] it is calculated that if 15% of the revised target of 1215 participants are included in the periodontitis sub-study, we will be able to estimate the difference in the mean percentage of periodontal sites at 6 months with PPD ≥ 5mm for VBA versus EC or NRT to within ± 4.4% and for EC vs NRT to within ± 3.6%.

The sample size calculation for the primary outcome was performed in SAS version 9.4 of the SAS System for Windows 7, copyright © 2012 SAS Institute Inc. using proc power two sample freq test=pchi. The sample size was verified in Stata 16 [12] using power two proportions test (chi2).

## 5. Oversight Committee composition

The IDMC and TSC are independent from the sponsor and must declare any potential competing interests. All members of the oversight committees sign their respective Charter which outlines their roles and responsibilities. The charter is available upon request.

**Independent data monitoring committee composition**

| **Name** | **Role and Institution** | **IDMC role** |
| --- | --- | --- |
| Dr Jamie Hartmann-Boyce | Associate Professor of Evidence-Based Policy and Practice, Oxford University, UK | Independent chair |
| Dr Lewis Winning | Associate Professor / Consultant in Restorative Dentistry, Dublin Dental University Hospital, UK | Independent member |
| Dr Rebecca Playle | Deputy Director of Statistics, Cardiff University, UK | Independent member |

**Trial oversight committee composition**

| **Name** | **Role and Institution** | **TSC role** |
| --- | --- | --- |
| Prof Caitlin Notley | Professor of Addiction Sciences, University of East Anglia, UK | Independent chair |
| Dr Gerry McKenna | Clinical Reader / Consultant in Restorative Dentistry, Queen’s University Belfast and Belfast Health and Social Care Trust, UK | Independent member |
| Prof Richard Watt | Chair and Honorary Consultant in Dental Public Health, University College London (UCL) and honorary contract with Central North West London NHS Foundation Trust, UK | Independent member |
| Prof Tanya Walsh | Professor of Healthcare Evaluation, Director of Research I Division of Dentistry I Faculty of Biology, Medicine and Health, University of Manchester, UK | Independent member |
| Dr Dwayne Boyers | Senior Research Fellow, Health Economics Research Unit, University of Aberdeen, UK | Independent member |
| Ms Juliet Schick | n/a | Independent member (lay member) |
| Mr Rashmi Kumar | n/a | Independent member (lay member) |
| Dr Richard Holliday | Clinical Senior Lecturer/Consultant Dental Sciences, Newcastle University, UK | Non independent member |
| Miss Chrissie Butcher | Senior Trial Manager, Newcastle University, UK | Non independent member |

## 6. Participant journey flow diagram

**
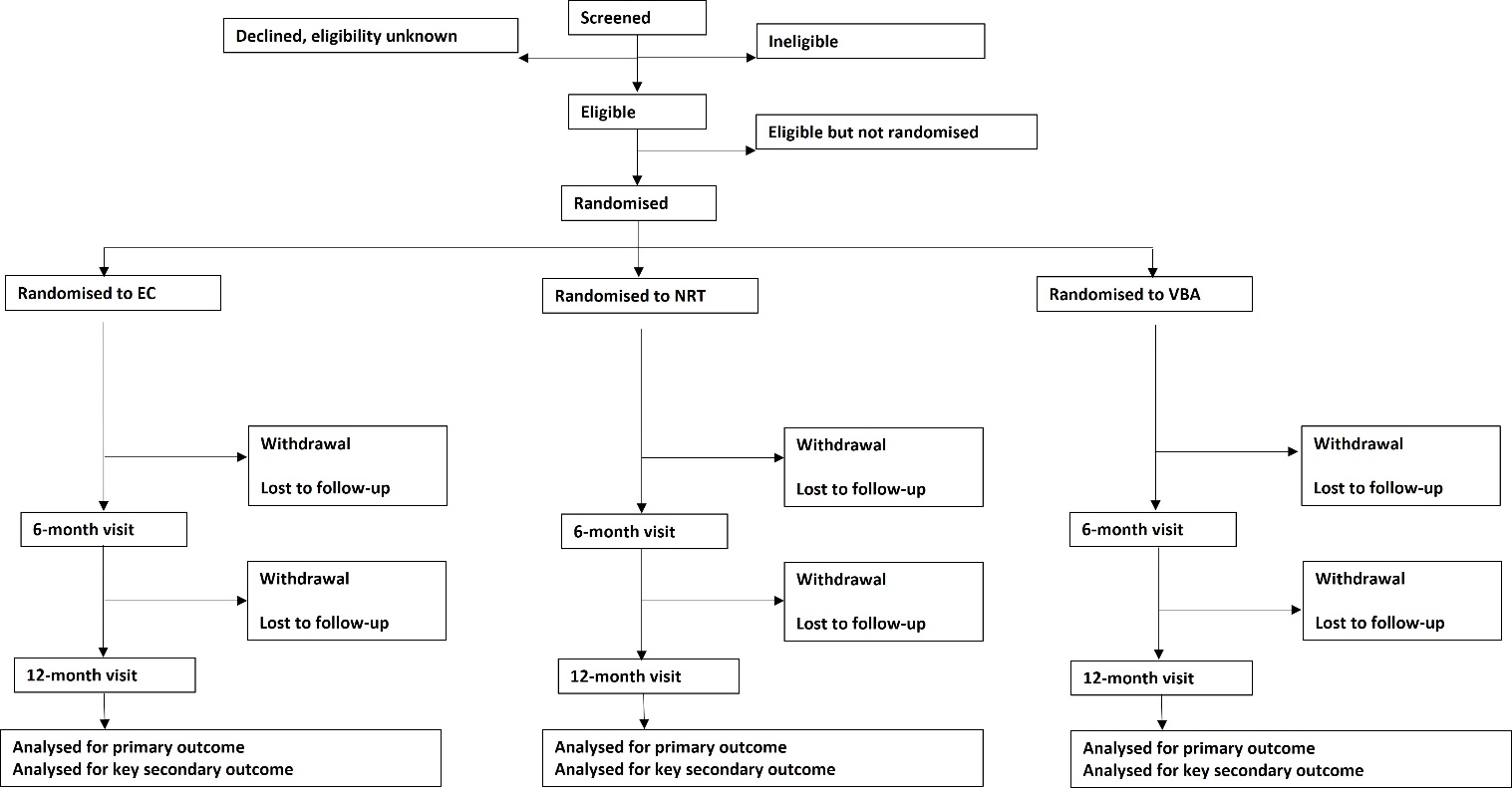
**

## 7. Additional file 1 references

1. Heatherton TF, Kozlowski LT, Frecker RC, Fagerstrom KO. The Fagerstrom Test for Nicotine Dependence: a revision of the Fagerstrom Tolerance Questionnaire. British journal of addiction. 1991;86(9):1119-27.

2. National Centre for Smoking Cessation and Training. Mood and Physical Symptoms Scale (MPSS). 2012.

3. Durham J, Fraser HM, McCracken GI, Stone KM, John MT, Preshaw PM. Impact of periodontitis on oral health-related quality of life. Journal of Dentistry. 2013;41(4):370-6.

4. McGrath C, Bedi R. Understanding the value of oral health to people in Britain--importance to life quality. Community dental health. 2002;19(4):211-4.

5. West R, Hajek P, Stead L, Stapleton J. Outcome criteria in smoking cessation trials: proposal for a common standard. Addiction (Abingdon, England). 2005;100(3):299-303.

6. Osailan SM, Pramanik R, Shirlaw P, Proctor GB, Challacombe SJ. Clinical assessment of oral dryness: development of a scoring system related to salivary flow and mucosal wetness. Oral surgery, oral medicine, oral pathology and oral radiology. 2012;114(5):597-603.

7. Lobene RR, Weatherford T, Ross NM, Lamm RA, Menaker L. A modified gingival index for use in clinical trials. Clinical preventive dentistry. 1986;8(1):3-6.

8. Silness J, Loe H. Periodontal disease in pregnancy. II. Correlation between oral hygiene and periodontal condition. Acta odontologica Scandinavica. 1964;22:121-35.

9. Nesse W, Abbas F, van der Ploeg I, Spijkervet FKL, Dijkstra PU, Vissink A. Periodontal inflamed surface area: quantifying inflammatory burden. Journal of Clinical Periodontology. 2008;35:668–73.

10. Food and Drug Administration. Multiple Endpoints in Clinical Trials Guidance for Industry. Food and Drug Administration; 2017.

11. Holliday R, Preshaw PM, Ryan V, Sniehotta FF, McDonald S, Bauld L, et al. A feasibility study with embedded pilot randomised controlled trial and process evaluation of electronic cigarettes for smoking cessation in patients with periodontitis. Pilot and Feasibility Studies. 2019;5(1):74.

12. StataCorp. Stata: Release 16. Statistical Software. College Station, TX: StataCorp LLC. 2019.
