# Supplementary material for: ENHANCE-D: protocol for a pragmatic, 3-arm, randomised controlled trial comparing the impact of enhanced smoking cessation interventions to very brief advice for adult smokers in dental care settings
Source: Trials. 2025 Jul 28;26:261. doi: 10.1186/s13063-025-08954-z (PMC12305899; doi:10.1186/s13063-025-08954-z)
Supplement: Supplementary file 2 — Additional file 2: SPIRIT checklist [file 13063_2025_8954_MOESM2_ESM.pdf]

SPIRIT Checklist for *Trials*

Complete this checklist by entering the page and line numbers where each of the items listed below can be found in your manuscript.

Your manuscript may not currently address all the items on the checklist. Please modify your text to include the missing information. If you are certain that an item does not apply, please state "n/a" and provide a short explanation. **Leaving an item blank or stating "n/a" without an explanation will lead to your manuscript being returned before review.**

Upload your completed checklist as an additional file when you submit to *Trials*. You must reference this additional file in the main text of your protocol submission. The completed SPIRIT figure must be included within the main body of the protocol text and can be downloaded here: http://www.spirit-statement.org/schedule-of-enrolment-interventions-and-assessments/

In your methods section, please state that you used the SPIRIT reporting guidelines, and cite them as:

Chan A-W, Tetzlaff JM, Gøtzsche PC, Altman DG, Mann H, Berlin J, Dickersin K, Hróbjartsson A, Schulz KF, Parulekar WR, Krleža-Jerić K, Laupacis A, Moher D. SPIRIT 2013 Explanation and Elaboration: Guidance for protocols of clinical trials. BMJ. 2013;346:e7586

		Reporting Item	Page and Line Number	Reason if not applicable
**Administrative information**				
Title	#1	Descriptive title identifying the study design, population, interventions, and, if applicable, trial acronym	P1, L6, 7	
Trial registration	#2a	Trial identifier and registry name. If not yet registered, name of intended registry	*P2, L29, 30*	
Trial registration: data set	#2b	All items from the World Health Organization Trial Registration Data Set	*Throughout whole paper, administrative aspects P18,L40 to P20,L6*	

Protocol version	#3	Date and version identifier	Under Trial status, P18, L41,42.	
Funding	#4	Sources and types of financial, material, and other support	P19, L27-30	
Roles and responsibilities: contributorship	#5a	Names, affiliations, and roles of protocol contributors	P1,L11-27 authors and affiliations and P18,L33-38 Authors' contributions	
Roles and responsibilities: sponsor contact information	#5b	Name and contact information for the trial sponsor	P19, L43-44.	
Roles and responsibilities: sponsor and funder	#5c	Role of study sponsor and funders, if any, in study design; collection, management, analysis, and interpretation of data; writing of the report; and the decision to submit the report for publication, including whether they will have ultimate authority over any of these activities	P20, L2-6	
Roles and responsibilities: committees	#5d	Composition, roles, and responsibilities of the coordinating centre, steering committee, endpoint adjudication committee, data management team, and other individuals or groups overseeing the trial, if applicable (see Item 21a for data monitoring committee)	Under Trial oversight committees and monitoring, P16 L 42 to P17 L12.	
**Introduction**			P3	
Background and rationale	#6a	Description of research question and justification for undertaking the trial, including summary of	P3, L3-30	

		relevant studies (published and unpublished) examining benefits and harms for each intervention		
Background and rationale: choice of comparators	#6b	Explanation for choice of comparators	P3, L12-25	
Objectives	#7	Specific objectives or hypotheses	P3, L31-42	
Trial design	#8	Description of trial design including type of trial (eg, parallel group, crossover, factorial, single group), allocation ratio, and framework (eg, superiority, equivalence, non-inferiority, exploratory)	P4, L6-10	
**Methods: Participants, interventions, and outcomes**				
Study setting	#9	Description of study settings (eg, community clinic, academic hospital) and list of countries where data will be collected. Reference to where list of study sites can be obtained	P4, L23-31	
Eligibility criteria	#10	Inclusion and exclusion criteria for participants. If applicable, eligibility criteria for study centres and individuals who will perform the interventions (eg, surgeons, psychotherapists)	P4, L33 to P5, L5.	
Interventions: description	#11a	Interventions for each group with sufficient detail to allow replication, including how and when they will be administered	P6, L11 to P7, L26. And table 1 on P8	
Interventions: modifications	#11b	Criteria for discontinuing or modifying allocated interventions for a given trial participant (eg, drug		Not applicable as the intervention is the 'offer' of an NRT or EC starter kit.

		dose change in response to harms, participant request, or improving / worsening disease)		
Interventions: adherance	#11c	Strategies to improve adherence to intervention protocols, and any procedures for monitoring adherence (eg, drug tablet return; laboratory tests)		There are no strategies to improve adherence to the trial interventions as the intervention is the 'offer' of an NRT or EC starter kit.
Interventions: concomitant care	#11d	Relevant concomitant care and interventions that are permitted or prohibited during the trial		Not relevant for our study design
Outcomes	#12	Primary, secondary, and other outcomes, including the specific measurement variable (eg, systolic blood pressure), analysis metric (eg, change from baseline, final value, time to event), method of aggregation (eg, median, proportion), and time point for each outcome. Explanation of the clinical relevance of chosen efficacy and harm outcomes is strongly recommended	Trial outcomes: P9, L1 to P10, L1<br><br>Justification of choice of AEs: P16, L30-40.	
Participant timeline	#13	Time schedule of enrolment, interventions (including any run-ins and washouts), assessments, and visits for participants. A schematic diagram is highly recommended (see Figure)	P11, Table 2	
Sample size	#14	Estimated number of participants needed to achieve study objectives and how it was determined, including clinical and statistical assumptions supporting any sample size calculations	P13, L1-15<br><br>Changes to original sample size: Additional file 1	

Recruitment	#15	Strategies for achieving adequate participant enrolment to reach target sample size	Recruitment: P5, L7-30	
**Methods: Assignment of interventions (for controlled trials)**				
Allocation: sequence generation	#16a	Method of generating the allocation sequence (eg, computer-generated random numbers), and list of any factors for stratification. To reduce predictability of a random sequence, details of any planned restriction (eg, blocking) should be provided in a separate document that is unavailable to those who enrol participants or assign interventions	P6, L3-10	
Allocation concealment mechanism	#16b	Mechanism of implementing the allocation sequence (eg, central telephone; sequentially numbered, opaque, sealed envelopes), describing any steps to conceal the sequence until interventions are assigned	P6, L3-10	
Allocation: implementation	#16c	Who will generate the allocation sequence, who will enrol participants, and who will assign participants to interventions	Generation: P6, L8-10<br><br>Enrolment: P5, L31-36 *'Who will take informed consent'*<br><br>Assignment: P6, L8-10	
Blinding (masking)	#17a	Who will be blinded after assignment to interventions (eg, trial participants, care providers, outcome assessors, data analysts), and how	P10, L3-24	

Blinding (masking): emergency unblinding	#17b	If blinded, circumstances under which unblinding is permissible, and procedure for revealing a participant's allocated intervention during the trial		Open label and so not applicable.
**Methods: Data collection, management, and analysis**				
Data collection plan	#18a	Plans for assessment and collection of outcome, baseline, and other trial data, including any related processes to promote data quality (eg, duplicate measurements, training of assessors) and a description of study instruments (eg, questionnaires, laboratory tests) along with their reliability and validity, if known. Reference to where data collection forms can be found, if not in the protocol	P10, L25 to P11, L4. Table 2 and Additional file 1.<br>Periodontal sub-study examiner alignment: P11, L7-23	
Data collection plan: retention	#18b	Plans to promote participant retention and complete follow-up, including list of any outcome data to be collected for participants who discontinue or deviate from intervention protocols	Retention: P10, L39 to P11,L2.<br>Remote eCO collection: P11, L1-2.	
Data management	#19	Plans for data entry, coding, security, and storage, including any related processes to promote data quality (eg, double data entry; range checks for data values). Reference to where details of data management procedures can be found, if not in the protocol	P10, L34-38	
Statistics: outcomes	#20a	Statistical methods for analysing primary and secondary outcomes. Reference to where other	P13, L17 to P14, L33.	

		details of the statistical analysis plan can be found, if not in the protocol		
Statistics: additional analyses	#20b	Methods for any additional analyses (eg, subgroup and adjusted analyses)	P14, L36-38	
Statistics: analysis population and missing data	#20c	Definition of analysis population relating to protocol non-adherence (eg, as randomised analysis), and any statistical methods to handle missing data (eg, multiple imputation)	Primary outcome: P14, L13-14.<br><br>Key secondary outcome: P14, L23-24	
**Methods: Monitoring**				
Data monitoring: formal committee	#21a	Composition of data monitoring committee (DMC); summary of its role and reporting structure; statement of whether it is independent from the sponsor and competing interests; and reference to where further details about its charter can be found, if not in the protocol. Alternatively, an explanation of why a DMC is not needed	P17, L1-12 and Additional file 1	
Data monitoring: interim analysis	#21b	Description of any interim analyses and stopping guidelines, including who will have access to these interim results and make the final decision to terminate the trial	P14, L35.	
Harms	#22	Plans for collecting, assessing, reporting, and managing solicited and spontaneously reported adverse events and other unintended effects of trial interventions or trial conduct	P16, L31-40	

Auditing	#23	Frequency and procedures for auditing trial conduct, if any, and whether the process will be independent from investigators and the sponsor	Onsite monitoring: P17, L5-7<br><br>Sponsor: P20, L3-6	
**Ethics and dissemination**				
Research ethics approval	#24	Plans for seeking research ethics committee / institutional review board (REC / IRB) approval	P19, L8-10	
Protocol amendments	#25	Plans for communicating important protocol modifications (eg, changes to eligibility criteria, outcomes, analyses) to relevant parties (eg, investigators, REC / IRBs, trial participants, trial registries, journals, regulators)	P17, L27-30	
Consent or assent	#26a	Who will obtain informed consent or assent from potential trial participants or authorised surrogates, and how (see Item 32)	P5, L32-42	
Consent or assent: ancillary studies	#26b	Additional consent provisions for collection and use of participant data and biological specimens in ancillary studies, if applicable	P5, L40-42	
Confidentiality	#27	How personal information about potential and enrolled participants will be collected, shared, and maintained in order to protect confidentiality before, during, and after the trial	P10, L28-30	
Declaration of interests	#28	Financial and other competing interests for principal investigators for the overall trial and each study site	P19, L25	

Data access	#29	Statement of who will have access to the final trial dataset, and disclosure of contractual agreements that limit such access for investigators	P19, L16-31	
Ancillary and post trial care	#30	Provisions, if any, for ancillary and post-trial care, and for compensation to those who suffer harm from trial participation		Not applicable, as the intervention is the 'offer' of an NRT or EC starter kit.
Dissemination policy: trial results	#31a	Plans for investigators and sponsor to communicate trial results to participants, healthcare professionals, the public, and other relevant groups (eg, via publication, reporting in results databases, or other data sharing arrangements), including any publication restrictions	P17, L33-37	
Dissemination policy: authorship	#31b	Authorship eligibility guidelines and any intended use of professional writers	Authors' contributions p19, L35-38	No intended use of professional authors
Dissemination policy: reproducible research	#31c	Plans, if any, for granting public access to the full protocol, participant-level dataset, and statistical code	P19, L16-21	
**Appendices**				
Informed consent materials	#32	Model consent form and other related documentation given to participants and authorised surrogates		PIS included in Additional file 1
Biological specimens	#33	Plans for collection, laboratory evaluation, and storage of biological specimens for genetic or	P9, L41	

		molecular analysis in the current trial and for future use in ancillary studies, if applicable		

It is strongly recommended that this checklist be read in conjunction with the SPIRIT 2013 Explanation & Elaboration for important clarification on the items. Amendments to the protocol should be tracked and dated. The SPIRIT checklist is copyrighted by the SPIRIT Group under the Creative Commons "Attribution-NonCommercial-NoDerivs 3.0 Unported" license. This checklist can be completed online using https://www.goodreports.org/, a tool made by the EQUATOR Network in collaboration with Penelope.ai
