# Supplementary material for: ENHANCE-D: protocol for a pragmatic, 3-arm, randomised controlled trial comparing the impact of enhanced smoking cessation interventions to very brief advice for adult smokers in dental care settings
Source: Trials. 2025 Jul 28;26:261. doi: 10.1186/s13063-025-08954-z (PMC12305899; doi:10.1186/s13063-025-08954-z)
Supplement: Supplementary file 3 — Additional file 3. Topic guides [file 13063_2025_8954_MOESM3_ESM.pdf]

ENHANCE-D: Qualitative Component

Method: Interviews/Focus Groups (in-person or online)

Topic Guide – PARTICIPANTS at 1 month post-intervention start

Background information for Qualitative Researcher:

- Thanks for participation
- Verbally *reconfirm former written consent* (held on file) & *reminder of audio-recording*
- Confidentiality**:** Participants' real names/practice names/dental professionals' names will not be used in the reporting of today's discussion. Comments made today may be used in the study team's formal report and in published research papers but in all cases your name and identity would remain confidential. It is important that the content of today's discussion is not shared with others after the interview/meeting has ended. The audio-recording will never be shared with anyone outside of the academic research team at Newcastle University.
- Aim of today's interview/focus group
- (*if Focus Group) We're interested to hear everyone's view so please feel free to add your comments and experiences (whether you agree or disagree with what you hear). However, only one person can talk at once to ensure a good quality audio-recording.
-There are no right or wrong answers – we'd like to hear about your thoughts, opinions & experiences in this study to date.
- I have a list of areas I'd like us to discuss today, that's why I may need to look at paperwork during the session and make a few notes.
- Any questions?

>TURN ON AUDIO-RECORDER (Begin with date/participant group & numbers attending)

Glossary:

EC = Electronic cigarette
NRT = Nicotine Replacement Therapy
VBA = Very Brief Advice (usual care).

DN = dental nurse
DH = dental hygienist
DT = dental therapist

1]. Introduction/s

Name/s of participant/s and UK region only. *Very briefly reconfirm the intervention/s received by patient/s in this study* (…this is a double-check & for clarity in later qualitative analysis – options are listed above in Glossary).

2]. Intervention Start *(within 1 week of intervention start only)*

- Looking back to when you FIRST received the intervention (EC/NRT/VBA) what were your initial thoughts about what you received? (Probe: Why/explain etc)

-Have you ever experienced the intervention you received before being recruited to this study? (Probe: thoughts/+ve/-ve/is this any different – in what way?)

- How did the dental professional discuss the intervention with you? (Probe: who did this/what was done well/what could have been done better? Time: sufficient/rushed/too long? Location in practice – privacy etc)

-Have you spoken or dealt with any other member of the dental team (other than the dentist) as part of this study? (e.g. dental nurse/hygienist/therapist)? Probe: In what way? / how did you find dealing with DN/DH/DT as opposed to the dentist?

-How did you feel about a dental professional offering you the intervention as opposed to someone outside of dentistry? (Probe: surprised?/comfortable? & explain why they think this)

- Within the first week after receiving the intervention (EC/NRT/VBA), how did you find it initially / how did you adapt to it during the early days? (Probe: Habits/benefits/challenges)

-If you encountered any issues or problems with EC/NRT/VBA in the first week or so - what were they/what did you do to overcome them & did they resolve the situation?

3]. Current experiences *(at time of interview)*

-Now, thinking of the present (as opposed to 1-week post-intervention) has your attitude changed towards the intervention given to you? (Probe: improved/stayed same/worsened – why?)

- How have you found adhering to the instructions given to you about your intervention? (Probe: level of adherence, ?missing days – please attempt to quantify if applicable, no longer using device/intervention – why not? / what could improve the situation for you?

-(?)Have you noticed any changes e.g. habits/symptoms linked to your oral or general health since beginning the intervention? (Probe: what are they/describe)

-What do you think about the frequency of monitoring of this intervention by your dental professional –Are you clear when you're next required to visit? (Probe: does this feel about right etc?)

-How do you feel about this initiative being managed and monitored by a NHS dental practice? (Probe: why / are there any benefits/disadvantages to a dental practice doing this Vs. non-dental settings?)

-What has been the best thing so far about your involvement in this research study about smoking cessation?

-And the worst thing so far being part of this study?

4]. Looking forwards *(future thoughts and potential expansion of SC in dentistry)*

- How convenient do you believe an NHS dental practice is for people as a setting for smoking cessation options in the future? (Probe: +ves/-ves & why do you think this?)

-Perceived benefits of dental practice settings for smoking cessation initiatives (accept from any perspective & beyond patients)

-Perceived challenges/barriers of dental practice settings for smoking cessation initiatives

-- Acceptability (if not explicitly covered previously) – how comfortable are you with dental professionals undertaking smoking cessation interventions? (Probe: where would you choose to access this type of service if you had a choice?)

-*Optional re. ACCESS TO HEALTHCARE* (after Penchansky & Thomas) x5 background areas of interest for the facilitator to consider when considering access to service:

affordability, (cost – and to whom?)

availability, (sufficient dental practices?)

accessibility, (getting to the service/practice re. geographic location)

accommodation, (building/estates and meeting the preferences/needs of different patients)

acceptability, (to patients etc)

- If NHS dental practices were able to offer this range of smoking cessation interventions in the future, do you think this would be attractive to dental patients (Probe: why/why not?)

- Can you identify any improvements or changes to what you've experienced so far in this study, that may improve your experience of smoking cessation options in dental practice? (What are they?)

-OTHER: Anything else we may have missed that anyone would like to add before we close this discussion?

Thank you for your comments. Your views will help us to better understand the views of patients and their experiences of smoking cessation interventions delivered in dental practice.

TURN OFF AUDIO-RECORDER

ENHANCE-D: Qualitative Component

Method: Interviews/Focus Groups (in-person or online)

Topic Guide – DENTAL PROFESSIONALS

Background information for Qualitative Researcher:

- Thanks for participation
- Verbally *reconfirm former written consent* (held on file) & *reminder of audio-recording*
- Confidentiality**:** Participants' real names/practice names/dental professionals' names will not be used in the reporting of today's discussion. Comments made today may be used in the study team's formal report and in published research papers but in all cases your name and identity would remain confidential. It is important that the content of today's discussion is not shared with others after the interview/meeting has ended. The audio-recording will never be shared with anyone outside of the academic research team at Newcastle University.
- Aim of today's interview/focus group
- (*if Focus Group) We're interested to hear everyone's view so please feel free to add your comments and experiences (whether you agree or disagree with what you hear). However, only one person can talk at once to ensure a good quality audio-recording.
-There are no right or wrong answers – we'd like to hear about your thoughts, opinions & experiences in this study to date.
- I have a list of areas I'd like us to discuss today, that's why I may need to look at paperwork during the session and make a few notes.
- Any questions?

>TURN ON AUDIO-RECORDER (Begin with date/participant group & numbers attending)

Glossary:

EC = Electronic cigarette
NRT = Nicotine Replacement Therapy
VBA = Very Brief Advice (usual care).

DN = dental nurse
DH = dental hygienist
DT = dental therapist

1]. Introduction/s

Name/s of participant/s and UK region only. *Very briefly reconfirm the breadth of experience of intervention/s delivered to patient/s in this study by these dental professionals* (…this is a double-check & for clarity in later qualitative analysis – options are listed above in Glossary).

2]. Intervention Start *(within 1 week of intervention start only)*

- Looking back to when you FIRST handed over or delivered the intervention with patients (EC/NRT/VBA) what were patient's *initial* thoughts about what they received? (Probe: +ves/-ve comments or concerns from patients, surprise, questioning as to why a DP is doing this, why/explain etc)

- How did you initially find communicating & explaining the intervention/s with the patient? (Probe: who did this in the practice/what was done well/what could have been done better? Time: sufficient/rushed/too long? Location in practice – privacy etc)

-How confident did you feel initially explaining and delivering the intervention/s to patients? (probe: why? Was your training sufficient? What went well/could change?)

-Who from the dental team (DN/DH/DT) has been involved with the intervention/s to date? (Probe: In what way? / Who has led it and why? / Would you have preferred other staff members to have been involved if you had the option – who and why?)

-How do you think patients have reacted to SC being delivered in a dental practice, opposed to other health care professionals outside of dentistry? (Probe: were any patients surprised?/comfortable? & explain why they think this is)

-Were there any early positive/beneficial comments you heard or were made aware of from patients re. the interventions as delivered?

-Have you encountered any early issues or problems from patients with EC/NRT/VBA in the first week or so (Probe: what were they/what did you do to overcome them & did they resolve the situation to the patient's satisfaction/any still ongoing?)

3]. Current experiences *(at time of interview)*

-Now, thinking of the present time (as opposed to 1-week post-intervention), has your professional behaviour or communication with patients changed as a result of the interventions you've been asked to deliver in this study? (Probe: What/how, time – duration of comms - improved/stayed same/worsened – why?)

-Which intervention/s is/are preferred by patients at this point – any early opinions? (Probe: which intervention appears most acceptable & least acceptable to patients & to the DP currently?)

- Have you patients reported being able to adhere to the instructions given to them about your intervention? (Probe: level of adherence, missing days – please attempt to quantify if applicable, no longer using device/intervention – why not? / what could improve the situation or have prevented it originally?

-What do you think about the frequency of your monitoring of this intervention – are you and your patients clear when next visit is required? (Probe: does this feel about right – why/why not?)

-How do you feel about these SC interventions being managed and monitored by NHS dental practices? (Probe: why / are there any benefits/disadvantages to a dental practice doing this Vs. non-dental settings?)

-How has the dental team adapted to delivering these SC interventions? (Probe: How? Could this be maintained by your team in the longer-term?)

-What has been the best thing so far about <u>your</u> (DP) involvement in this research study about smoking cessation?

-And the worst thing so far being part of this study?

4]. Looking forwards *(future thoughts and potential expansion of SC in dentistry)*

-If as a DP had a free choice, which of the SC interventions would you choose to deliver in the future (Probe: Why?) Which if any, would you not choose to deliver (Probe: Why?)

- How convenient do you believe an NHS dental practice is for people as a setting for smoking cessation options in the future? (Probe: +ves/-ves & why do you think this?)

-What about NHS dental contractual/financial considerations for SC interventions in the future? (Probe: +ves/-ves, availability and sustainability of supplies, costs, contract, effect on waiting lists etc)

-What are the benefits of dental practice settings delivering smoking cessation initiatives (accept from any perspective e.g. NHS/taxpayer/patient/dental principal/contract holder/commissioner)

-Perceived challenges/barriers of dental practice settings for smoking cessation initiatives

-- Acceptability (if not explicitly covered previously) – how comfortable are you as a dental professionals undertaking smoking cessation interventions? (Probe: Acceptability to patients, to them as DPs, to other health care professionals, political acceptability?)

-*Optional re. ACCESS TO HEALTHCARE* (after Penchansky & Thomas) x5 background areas of interest for the facilitator to consider when considering access to service:

affordability, (cost – and to whom?)

availability, (sufficient dental practices?)

accessibility, (getting to the service/practice re. geographic location)

accommodation, (building/estates and meeting the preferences/needs of different patients)

acceptability, (to patients etc)

- If NHS dental practices were able to offer this range of smoking cessation interventions in the future, do you think this would be attractive to dental patients (Probe: why/why not?)

- Can you identify any improvements or changes to what you've experienced so far in this study, that may improve your experience of smoking cessation options in dental practice? (What are they?)

-OTHER: Anything else we may have missed that anyone would like to add before we close this discussion?

Thank you for your comments. Your views will help us to better understand the views of patients and their experiences of smoking cessation interventions delivered in dental practice.

TURN OFF AUDIO-RECORDER

ENHANCE-D TOPIC GUIDE

FOR NHS DENTAL COMMISSIONERS / POLICY LEADS

Introduction

- Thanks for participation and verbal consent for audio-recording.
- Reassurance of confidentiality and anonymity. State Importance of participant keeping discussion confidential.
- Aim of interview- THOUGHTS, OPINIONS & EXPERIENCES of Dental Professionals in delivering the interventions in this study to date. No right or wrong answers.
- Any questions?
- TURN ON AUDIO-RECORDER
- Date & time > Participant's Name > Participant's Region> Confirm Participant's role in the study

>>

Conclusion

- OTHER: Anything else we may have missed that you/anyone would like to add before we close this discussion?

Thank you for your comments. Your views will help us to better understand the experiences of Dental Professionals in delivering the intervention in dental practice. TURN OFF AUDIO-RECORDER.

QUESTIONS

**Topic**	**Area**	**Probes**
Future suitability of Dental professionals for intervention	Potential DPs to be involved in intervention at dental practices	Which DPs? Why?<br>Ideal role of DPs mentioned? Why?<br>Who should have oversight responsibility at practice? Why?<br>Would non-professional staffs be involved in practice? Which ones? Why? Role?
	Training of DP to give intervention	Compulsory for all or optional? Any DP in particular? Why?<br>Training incentivised? Why? How?<br>Refresher training? How often? CPD points?<br>How should it be executed/ managed in practice? Why?
	Dental team adapting to delivering these interventions	How? Could this be maintained by dental teams in the longer-term?<br>Potential additional adaptative measures to be seen? Which ones? Why?
	Comfortability undertaking such interventions	Acceptable to DPs? Acceptable to other health professionals?<br>Political acceptability?
	Best thing about DPs involvement in this research study	Why? Explain
Future suitability of dental practice setting for intervention	NHS dental practices managing and monitoring intervention	Why? - benefits/disadvantages to a dental practice doing this Vs. non-dental settings?
	Convenience of NHS dental practice for such intervention in the future	+ves/-ves? - why do you think this?
	Accessibility of dental practices	Sufficient dental practices? Potential barriers to accessibility? Measures to improve accessibility?
	NHS dental contractual/financial considerations for such interventions in the future	+ves/-ves? - availability and sustainability of supplies? Costs? Contract?<br>Effect on waiting lists?
	Benefits of dental practice settings delivering such interventions	How? Explain
	Perceived challenges/barriers of dental practice settings for such interventions	Why? Explain
Logistics of executing intervention in dental practice	Financing of interventions	Who should pay for interventions, E.g., patient, NHS, local council? Why? Explain<br>How might funding be executed?
	Prescription and ordering arrangement for interventions	How? Why? Explain
